# Large-scale micron-order 3D surface correlative chemical imaging of ancient Roman concrete

**DOI:** 10.1371/journal.pone.0210710

**Published:** 2019-02-06

**Authors:** Janille M. Maragh, James C. Weaver, Admir Masic

**Affiliations:** 1 Department of Civil and Environmental Engineering, Massachusetts Institute of Technology, Cambridge, Massachusetts, United States of America; 2 Wyss Institute for Biologically Inspired Engineering, Harvard University, Boston, Massachusetts, United States of America; University of Freiburg, GERMANY

## Abstract

There has been significant progress in recent years aimed at the development of new analytical techniques for investigating structure-function relationships in hierarchically ordered materials. Inspired by these technological advances and the potential for applying these approaches to the study of construction materials from antiquity, we present a new set of high throughput characterization tools for investigating ancient Roman concrete, which like many ancient construction materials, exhibits compositional heterogeneity and structural complexity across multiple length scales. The detailed characterization of ancient Roman concrete at each of these scales is important for understanding its mechanics, resilience, degradation pathways, and for making informed decisions regarding its preservation. In this multi-scale characterization investigation of ancient Roman concrete samples collected from the ancient city of Privernum (Priverno, Italy), cm-scale maps with micron-scale features were collected using multi-detector energy dispersive spectroscopy (EDS) and confocal Raman microscopy on both polished cross-sections and topographically complex fracture surfaces to extract both bulk and surface information. Raman spectroscopy was used for chemical profiling and phase characterization, and data collected using EDS was used to construct ternary diagrams to supplement our understanding of the different phases. We also present a methodology for correlating data collected using different techniques on the same sample at different orientations, which shows remarkable potential in using complementary characterization approaches in the study of heterogeneous materials with complex surface topographies.

## Introduction

Throughout human history, the development of materials processing technologies, supported by the existence of biological and geological materials with favorable mechanical properties, has played a key role in the cultural evolution of our species. Bones had been cut and polished in the production of ancient cutting and grinding tools since at least the Upper Paleolithic [1]. From 4000 BCE to the Late Bronze Age in Europe and Western Asia, and from about 800 to 1450 CE in Andean zone of South America, copper was alloyed with arsenic to produce arsenical bronze, which was both stronger and had better casting properties than copper alone [2]. Iron has been alloyed with carbon to produce steel since at least 1800 BCE, as indicated by the discoveries of ancient steel artifacts in Kaman-Kalehöyük [3]. Developments in construction include the production of lime cements, and later the incorporation of volcanic ash to produce high strength cements [4], for example in the production of hydraulic mortar using ash obtained from the area around Mount Vesuvius in ancient Rome [5]. The development of materials and building technologies and their empirical improvements have made significant contributions to the success of a number of ancient empires [6]. As an example, the growing population of the Roman Empire was sustainable for centuries, partially due to the construction of aqueducts that transported water from springs that were dozens of kilometers away [7,8]. The sophistication of some of these ancient building techniques is observable in the enduring remains of ancient civilizations in the present day, some of which still exhibit durability despite millennia of seismic activity, environmental changes, and natural disasters [9,10]. In addition to their cultural and historical significance, these materials may also offer modern researchers technological lessons in terms of sustainability and durability. The long-term resistance of ancient Roman concrete to environmental degradation over the course of millennia, for example, could provide design inspiration in the production of a new generation of more durable construction materials.

The use of ancient Roman cement in the production of mortar (mixture of cement paste and fine aggregate) and *opus cæmenticum* or ancient Roman concrete (mixture of mortar with larger aggregate: *cæmenta*) [11] permitted the fabrication of some of antiquity’s most massive architectural marvels. The use of lime in the production of cement was not original to the Roman Empire; the Greeks had previously produced cement in this manner with varying levels of success. However, the addition of *pozzolana* (high alkali volcanic ash, primarily consisting of amorphous aluminosilicates), native to the areas surrounding Mount Vesuvius in Campania, to this mixture resulted in a superior binding mortar [5,12], which produced concretes with a greater compressive strength than concrete produced using pure lime cement. Replicas of architectural ancient Roman mortar have been found to have a 180-day compressive strength of approximately 13 MPa [13], which is 75% of that of modern structural lightweight aggregate concrete [14], and at least four times greater than that of pure lime mortar [15].

Ancient Roman cement paste is a mixture of slaked lime and siliceous volcanic ash (*pozzolana*). In its production process, calcium carbonate (limestone) is calcined at 900°C to produce calcium oxide, which is then mixed with water to produce slaked lime. Amorphous or poorly-crystalline calcium silicate hydrate (C-S-H) and calcium aluminum silicate hydrate (C-A-S-H) gel phases are then produced in reaction layers surrounding the *pozzolana* fragments as they react with the slaked lime [16], [17,18]; these gel phases significantly contribute to the durability of ancient Roman mortar and its resistance to environmental damage [18]. Furthermore, recent studies have shown that mineral cements may be continually precipitated over long periods of time, continually reinforcing the cementitious matrix, in certain concretes, such as ancient Roman seawater concrete, [19]. This phenomenon stands in stark contrast to the problem of the alkali silica reaction (ASR), also known as “concrete cancer”, which causes the swelling and cracking of modern concrete due to the reaction of the highly alkaline cementitious matrix with siliceous aggregates in the presence of moisture. Comprehensive studies of the chemical mechanisms in such ancient concretes are imperative in the understanding of cements that are able generate new cementitious phases over time, which may prove to be crucial in the design of new construction materials.

The production of the world’s mostly widely used modern construction material, ordinary Portland cement (OPC), accounts for approximately 5% of the world’s carbon emissions [20], and 90% of its embodied carbon is due to the large fuel requirement for its production, particularly the hot stage, which occurs at ca. 1450°C. The significantly lower temperatures required for the production of ancient Roman concrete equate to a lower fuel requirement: another reason to look to this material for inspiration in the design of more sustainably produced construction materials. Ancient Roman concrete does, however, exhibit some significant drawbacks. Whereas cement paste made using OPC may cure in air in as little as 28 days to achieve a compressive strength close to its maximum [21], the volcanic ash lime-based architectural ancient Roman mortar has been shown to require a comparatively longer cure time of at least 180 days [22], making it much less viable for use in modern fast-paced construction environments. However, by partially replacing the constituents of modern concrete with components inspired by these ancient Roman concrete mixtures, an improved cement that balances durability and sustainability with an acceptable curing time could be developed. To set the stage for this process and to gain a better understating of the compositional complexity of this heterogeneous material, we investigated samples of ancient Roman concrete through the use of complementary advanced materials characterization techniques.

OPC consists of two major components: alite (Ca_3_SiO_5_ or “C_3_S” in cement chemistry notation), primarily responsible for the 28-day “early” strength of hydrated cement, and belite (Ca_2_SiO_4_ or “C_2_S”), primarily responsible for the development of its “late” strength [16]. Confocal Raman microscopy (CRM), which uses a Raman spectrometer in conjunction with an optical microscope to enable the measurement of very small areas, is one such method for the analysis of these specific phases and has been used in modern cement chemistry research to study the microstructure and spatial distribution of C-S-H and other reactants formed in hydrating C_3_S in a non-destructive manner [23]. This technique can also supplement more labor-intensive and time consuming methods, such as ^29^Si nuclear magnetic resonance (NMR) spectroscopy, in the characterization of C-S-H, C-A-S-H and sodium and aluminum substituted C-S-H (C-N-A-S-H) phases [24]. Raman spectroscopy has also been used in the study of degradation processes in OPC, through the spatial characterization of phases that cause a reduction in cement hardening in C_3_S and C_2_S pastes [25]. In addition to Raman-based techniques, scanning electron microscopy (SEM) based characterization approaches such as energy dispersive spectroscopy (EDS), which can be used for the identification and spatial mapping of elemental ratios, have been used in the characterization of hydrated OPC and volcanic ash mixtures and for the production of CaO-SiO_2_-Al_2_O_3_ ternary phase diagrams, which can be used to identify specific C-A-S-H phases [26,27]. When the ternary ratios of particular oxides are plotted, either for OPC or in the characterization of ancient Roman concrete [4,28], the proportion of particular gels and other phases may be determined, and specific components may be identified. When large area EDS maps are collected for a given sample, the vast amounts of data collected (often millions of spectra) can be used to compute these ternary ratios in a much more comprehensive fashion as a ternary frequency plot, on which chemically distinct components of a heterogeneous sample may be identified [29].

Whereas other characterization techniques introduce inconvenient challenges, such as the need for ultrathin section preparation as is the case of transmission electron microscopy, the lack of spatial resolution of detected phases in bench-top X-ray diffraction, or the low contrast between different cement phases typically seen in SEM/BSE imaging data [30,31], CRM and EDS mapping are appealing choices due to their need for minimal sample preparation and their utility in spatially resolving phases and elements present in a sample, with high compositional specificity.

The high-throughput nature of EDS means that one can quickly determine the ratios and spatial distributions of different elements, and for CRM, Raman active phases only need to be in focus to be detected. When both techniques are utilized in parallel, the result is a rich set of complementary data, but each approach is not without its limitations: EDS yields the distribution of elements everywhere on the surface of a sample measured, and the development of environmental SEMs over the past several decades allow for sample measurement even when not under vacuum conditions. However, measuring submerged samples using EDS remains a challenge. CRM, in contrast, may be used to detect the phases present in samples even when underwater when a water dipping objective is used, but only in cases where a Raman active species is present.

CRM and EDS have both been employed in the study of ancient concretes, for example in the characterization of ancient Roman seawater concrete, as tools in detecting precipitated phillipsite, Al-tobermorite, and gel-like C-A-S-H in relict lime clasts and in interfacial zones along the perimeter of certain aggregates, which are believed to contribute to the resilience of the concrete [9,19]. EDS has also been used to study ancient pozzolanic cements containing strätlingite, a mineral that reinforces the cementitious matrix and grows as plates that resist crack propagation [10], which have been detected in ancient Roman mortar found in the Colosseum [32], the Theatre of Marcellus [33], and in the wall core of the Great Hall of Trajan’s Markets [34].

These characterization techniques have been developed for use in the field of biomineralization to gain a better understanding of the complex structure-function relationship of heterogeneous mineralized tissues. EDS and CRM, for example, have been optimized to enable the collection of micron-resolution phase maps of polished sections of mantis shrimp appendages [35] and the brick-and-mortar nanostructures of mollusk nacre [6,36]. These techniques have also been augmented for use on irregular sample surfaces. With the development of *TrueSurface* profilometry, it has become possible to pair large area CRM with topography measurements through the use of a piezo-driven stages to maintain confocality. EDS systems have also been developed with multiple detectors to largely eliminate shadowing artifacts, which are frequently a problem encountered when acquiring element maps from irregular surfaces with a single X-ray detector. By harnessing the combined strengths of multi-detector EDS and *TrueSurface* Raman spectroscopy in the study of topographically complex sea urchin teeth, for example, the element distribution of dried sample surfaces could be measured either using EDS under vacuum, or in the case of native hydrated samples, the location of the Raman v_1_ CO_3_^2-^ could be used for measuring the extent of Mg substitution in the calcite crystal lattice [37].

In the present study, these two techniques are used to characterize ancient Roman concrete samples obtained from the Privernum archaeological site in Priverno, Italy. An approach for correlating data from these two techniques, in which a transformation matrix that relates the two datasets is computed using their corresponding phase maps, is also presented. Quantitative EDS maps were collected from both ancient Roman concrete cross-sections and fracture surfaces, and the ratios of the resulting elemental compositions were then visualized using ternary density plots, which demonstrated distinct compositional differences.

## Correlative data acquisition

**Fig 1:**
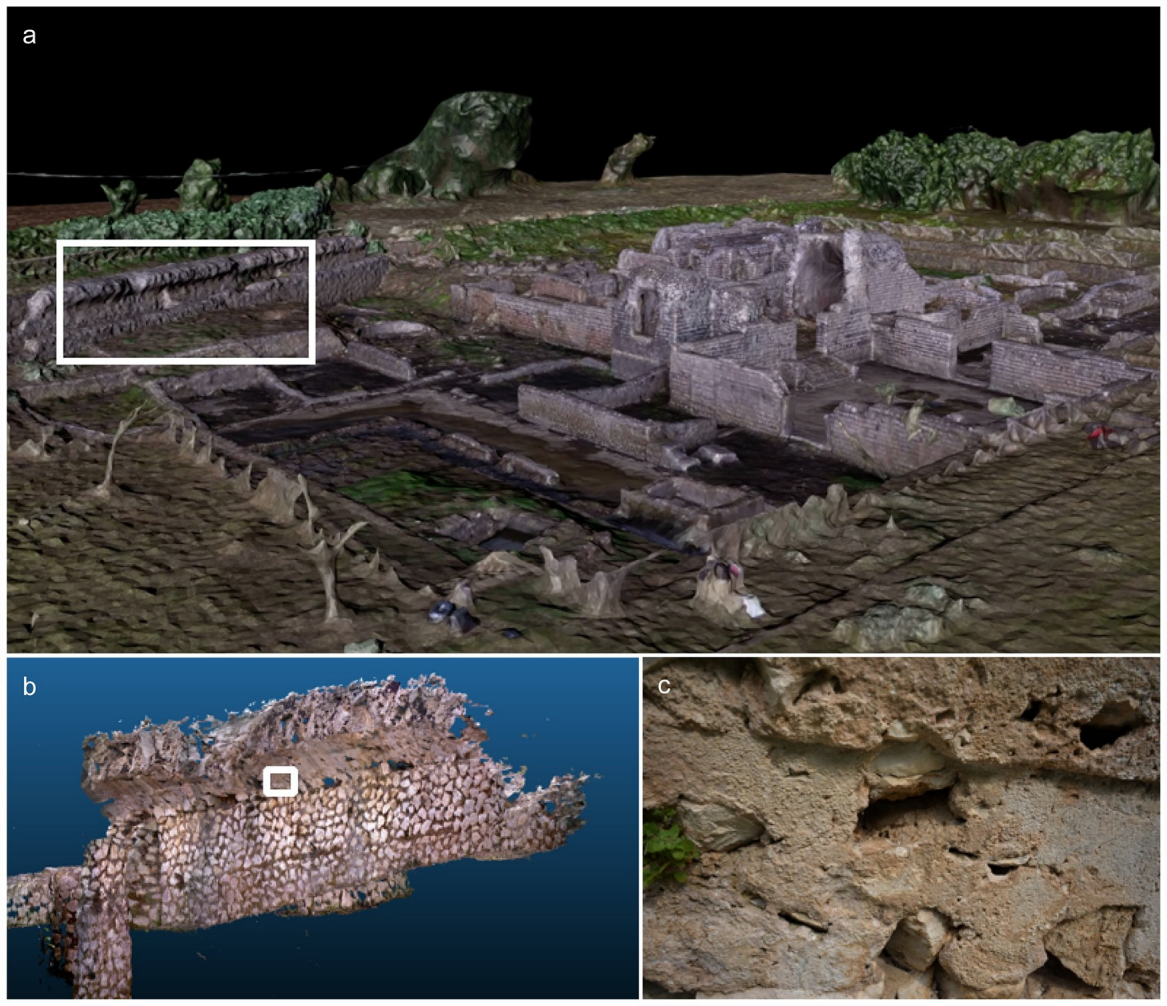
Collection location of the ancient Roman concrete samples used in this study. (a) 3D reconstruction of the Privernum archaeological site, collected using drone photogrammetry, (b) 3D reconstruction of the wall from which the concrete sample was collected, using a DotProduct DPI-8 3D scanner, and (c) a higher magnification photograph of the wall surface.

This study presents the chemical characterization of ancient Roman concrete samples sourced from a perimeter wall (Fig 1b and 1c) of the archaeological site (3D reconstruction of the site shown in Fig 1a) of Privernum, located in the modern city of Priverno, Italy, and introduces data management tools for correlating large amounts of complementary data that may be used in the validation of reproductions of this ancient mix and in the study of newly designed construction materials.

**Fig 2:**
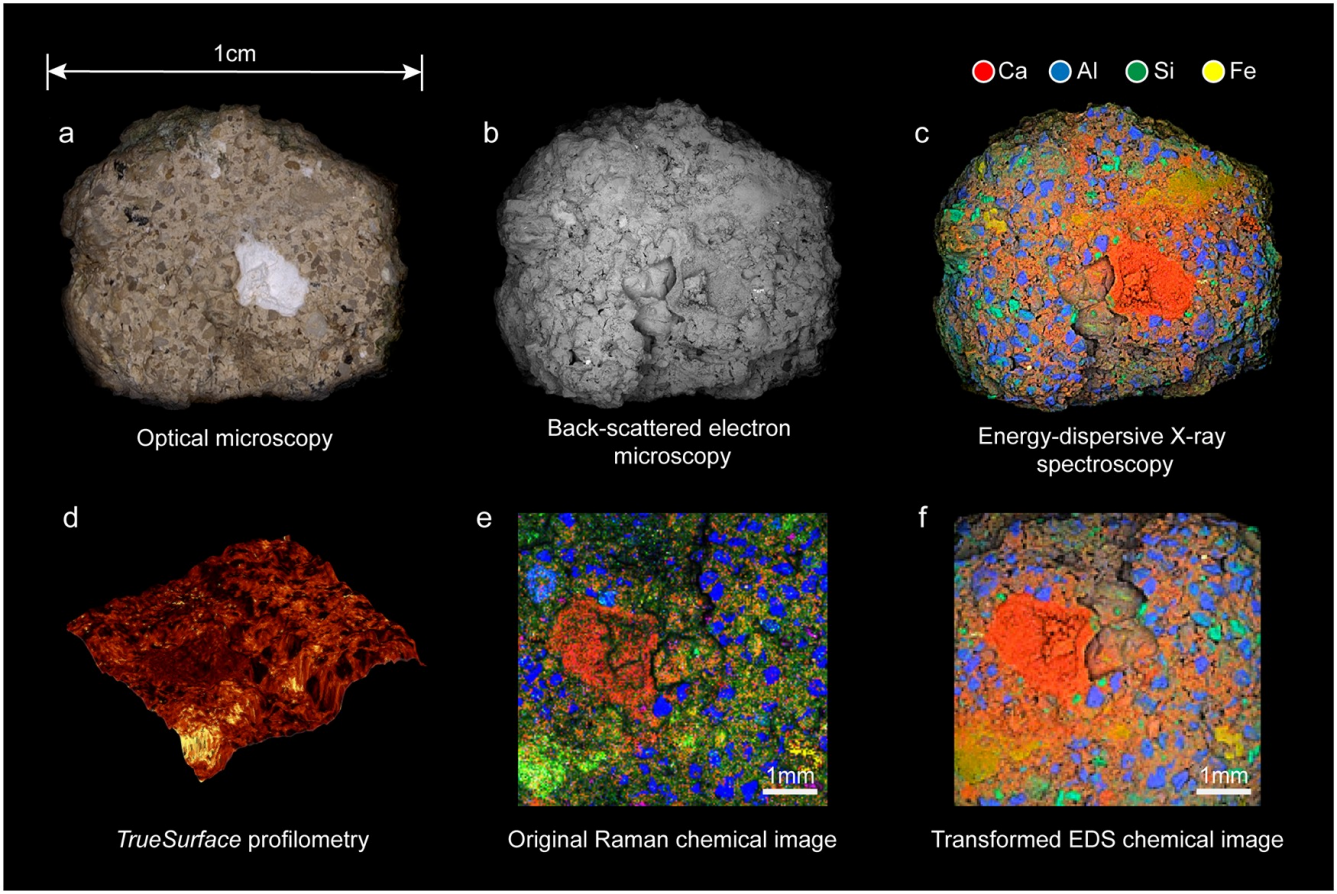
Multi-scale characterization of ancient Roman concrete. Images obtained using (a) optical microscopy, (b) backscattered scanning electron microscopy (BS-SEM), (c) energy dispersive spectroscopy (EDS) and (d) topography of a sub-region of concrete sample obtained using *TrueSurface* profilometry, (e) Raman phase map of that region (blue: quartz, red: calcite) and (f) EDS element map transformed into that region’s coordinate system.

Fractured ancient Roman concrete samples were studied using optical microscopy, backscattered scanning electron microscopy (BS-SEM), EDS and CRM coupled with *TrueSurface* profilometry. By leaving the samples in their native state, the fracture surfaces could be directly investigated, which is important in studying the cementitious binding phase, present in much greater quantities on the fracture surface than in polished cross sections. The samples were of sizes ranging from several millimeters to approximately 1 centimeter in width and Fig 2 shows the data collected for one particular sample collected from the wall section shown in Fig 1 using the aforementioned techniques.

The normalized distribution of elements on the sample’s fracture surface was measured using multi-detector EDS (Fig 2b). After measuring the topography of the sample using profilometry (Fig 2d), individual phases were extracted from the total set of Raman spectra collected using non-negative matrix factorization (NMF), which were then replotted to form the Raman phase map (Fig 2e).

**Fig 3:**
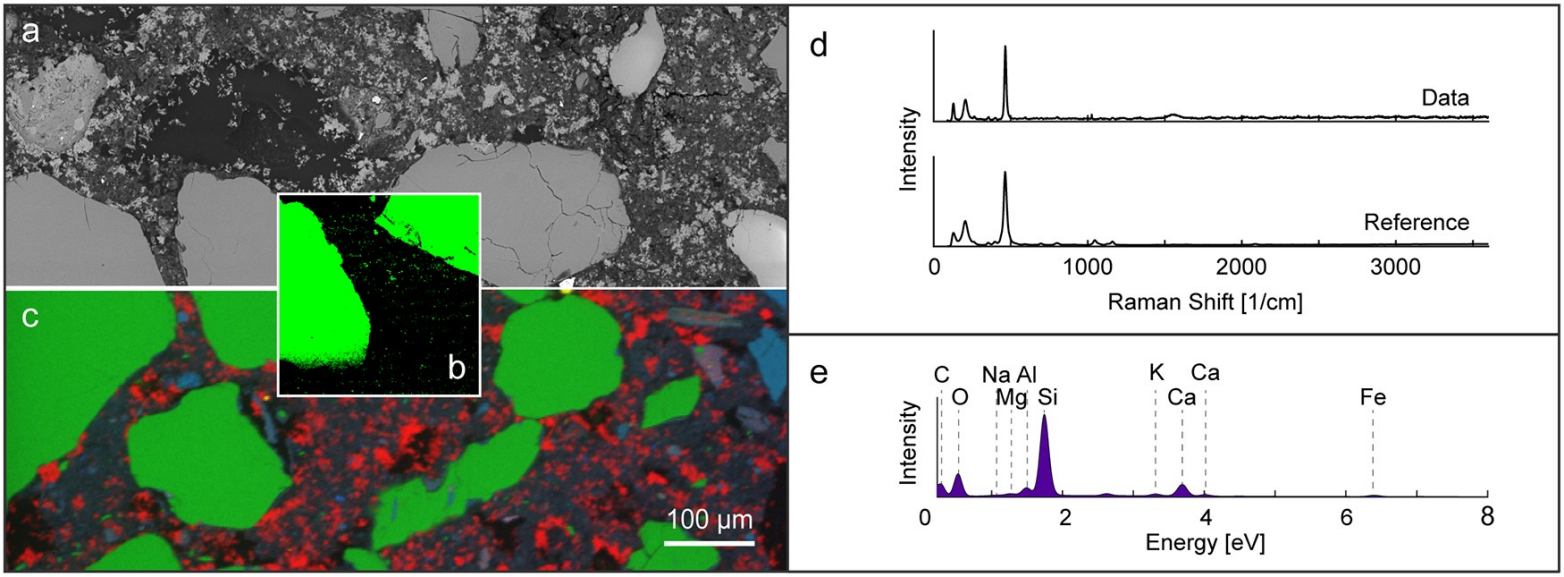
Correlative BS-SEM, EDS and Raman phase mapping conducted using an integrated system. (a) (BS-SEM image, (b) EDS map, (c) Raman phase map showing quartz distribution, (d) Raman spectrum from individual quartz grains and a reference quartz spectrum from the RRUFF database, and (e) an EDS spectrum form the entire mapped region.

When different types of data are collected on a single measurement system using the same frame of reference, they can be directly compared, as shown in Fig 3. In this particular system, a polished sample may remain in place, and SEM images, EDS element maps, and Raman phase maps may be collected and then later scaled for comparison.

However, when the sample orientation and measured area vary from dataset to dataset, as is often the case when multiple measurement systems are used, as also illustrated in this study, the task of comparing different types of data at the same location on a sample can become challenging. For large regions, such as the calcium-rich inclusion near the center of Fig 2c, the comparison between Raman data and EDS data is trivial, but the difficulty of this task grows with the size of the area being measured and the compositional complexity of the sample. All-in-one correlative systems, such as the system used to collect the data shown in Fig 3, are extremely useful, but may be insufficient when specific challenges arise. A general approach to correlating data would be needed, for example, if the measured sample has an irregular surface, if phase maps larger than what a combined system may produce are needed, or if pixel to pixel comparison of raw data is desired.

### Correlating EDS and Raman data

The Raman phase map in Fig 2e shows a different measured area and orientation than the BS-SEM and EDS results seen in Fig 2a, 2b and 2c. Different views of the same three-dimensional object are related to each other by a projective transformation known as planar homography: a 3x3 transformation matrix that relates the two projective spaces. Thus, data from the phase maps obtained using the two measurement systems, and thus the two sample orientations, can be correlated by computing the homography matrix, *H*, that relates them. The resulting transformation, when applied to the EDS dataset, results in the element map shown in Fig 2f, and it enables the direct pixel-to-pixel comparison of data represented by the complementary distribution maps.

To carry out this transformation, the Raman phase map was first resized so that the number of pixels corresponded to the number of Raman spectra encoded in the phase map, i.e. to 200 pixels by 200 pixels, representing the 40,000 spectra recorded. The color at each pixel of the EDS map was determined by the spectrum recorded at that sample location, so the number of pixels already corresponded with its number of spectra: 512 pixels by 512 pixels. The 8 unknowns, *h*_*ij*_, of the 3x3 homography matrix, *H*, (defined in Eq 1) may be solved for using the x- and y-coordinates of four corresponding locations on each phase map. The relationship between the coordinates in the two phase maps is shown in Eq 2, in which (x, y) are the coordinates of a point in the image to be transformed and (x’, y’) are the coordinates of the corresponding point in the second image.

$$\text{H}=\left[\begin{array}{ccc} H_{11} & H_{12} & H_{13}\\ H_{21} & H_{22} & H_{23}\\ H_{31} & H_{32} & 1 \end{array}\right]$$ (1)

$$\left[\begin{array}{c} x^{\prime }\\ y^{\prime }\\ 1 \end{array}]\sim H[\begin{array}{c} x\\ y\\ 1 \end{array}\right]$$ (2)

The *homography* function from the Machine Vision Toolbox [38] was used to compute the components of *H* representing the 2D projective transformation, using several corresponding locations from the two datasets. This transformation matrix was them applied to transform the EDS element maps into the same projective space as the corresponding Raman phase maps.

## Results and discussion

### Polished ancient Roman concrete cross-sections

**Fig 4:**
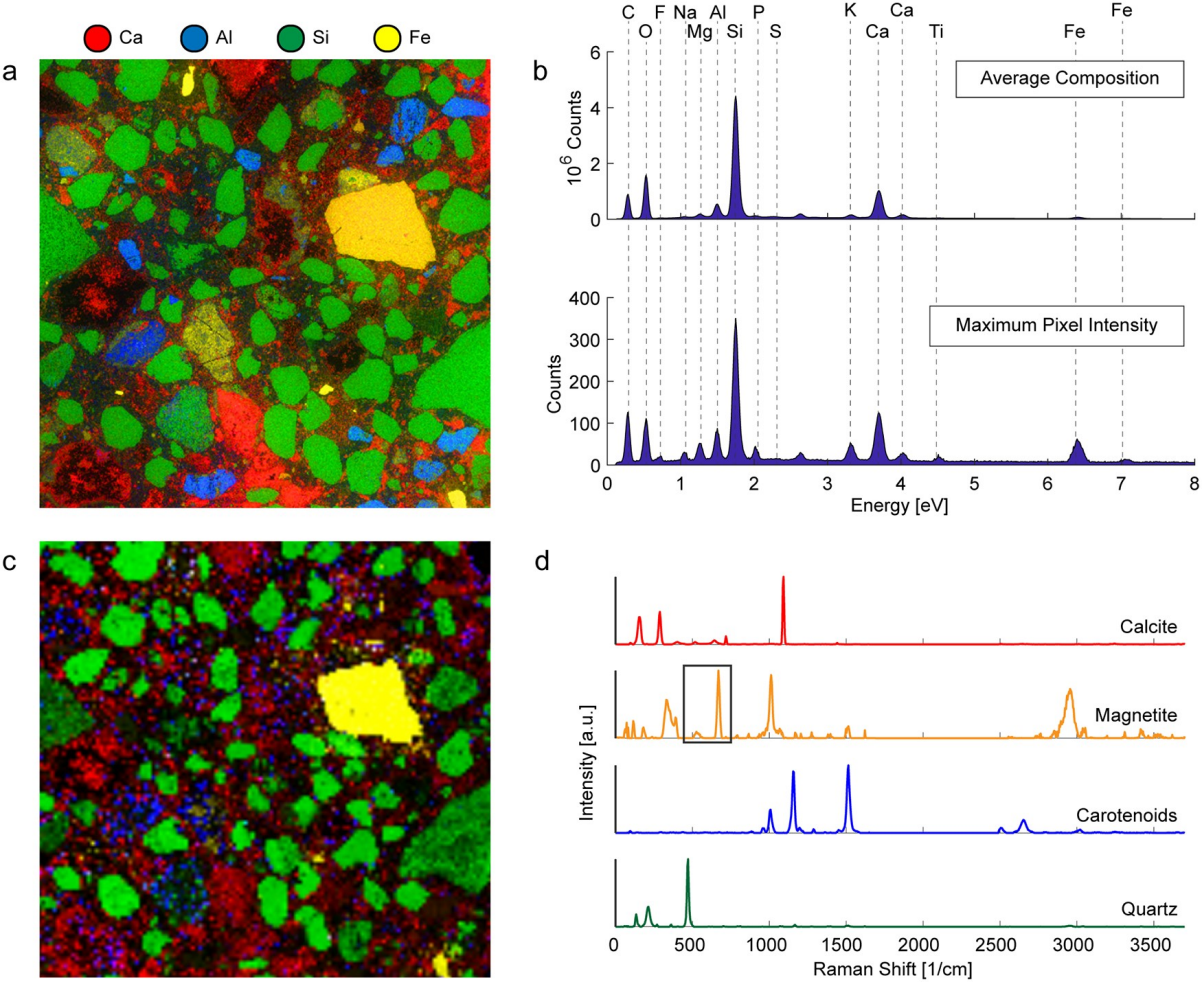
Correlative EDS and Raman characterization of a polished thin section of ancient Roman concrete. (a) EDS element map of polished concrete sample and (b) EDS spectra of shown sample area indicating the total average composition of the sample (upper) and the combined maximum pixel intensities of the detected elements (lower). (c) Raman phase map of polished thin-section showing region measured using EDS and spatial distribution of (d) the constituent spectra (which are color-coded corresponding to the different displayed map phases).

In order to study the average composition of the samples collected from the archaeological site, EDS element maps (Fig 4a) and Raman phase maps (Fig 4c) of polished thin-sections of ancient Roman concrete samples were collected. The total average EDS spectrum, shown in Fig 4b, which shows the total number of X-rays emitted and their energies, describes the average elemental composition of the measured area. The maximum pixel intensity spectrum, which shows the maximum number of counts for a particular X-ray energy detected anywhere in the sample (which is especially useful for the identification of trace elements), is shown in Fig 4b. The Raman spectra of the phases detected, extracted from the entire dataset using NMF, are shown in Fig 4d in colors corresponding to the colors used in the Raman phase map in Fig 4c.

Silicon-rich areas shown in green in Fig 4a correspond to the grains colored green in Fig 4c, which were identified as quartz, a major component of the siliceous sand used to produce ancient Roman mortar. The calcium-rich areas in Fig 4a correspond to the calcite-rich regions shown in Fig 4c, and the iron-rich regions, colored yellow in both Fig 4a and 4c, were identified from the Raman spectra to be magnetite. The region along the perimeter of the pore in the top-right corner and other regions colored blue in Fig 4c were found to consist of carotenoids, organic pigments produced by plants and algae, and some bacteria and fungi. The presence of carotenoids is likely due to the exposure of the archaeological ruins to the environment. The cementitious binding phase in the regions between the aggregate yielded Raman spectra with large amounts of background fluorescence that obscured any detectable Raman signal. This is often an issue when a material contains features that absorb laser radiation, such as volcanic ash containing rare earth elements: a known component of ancient Roman cement.

### Fracture surface of ancient Roman concrete

**Fig 5:**
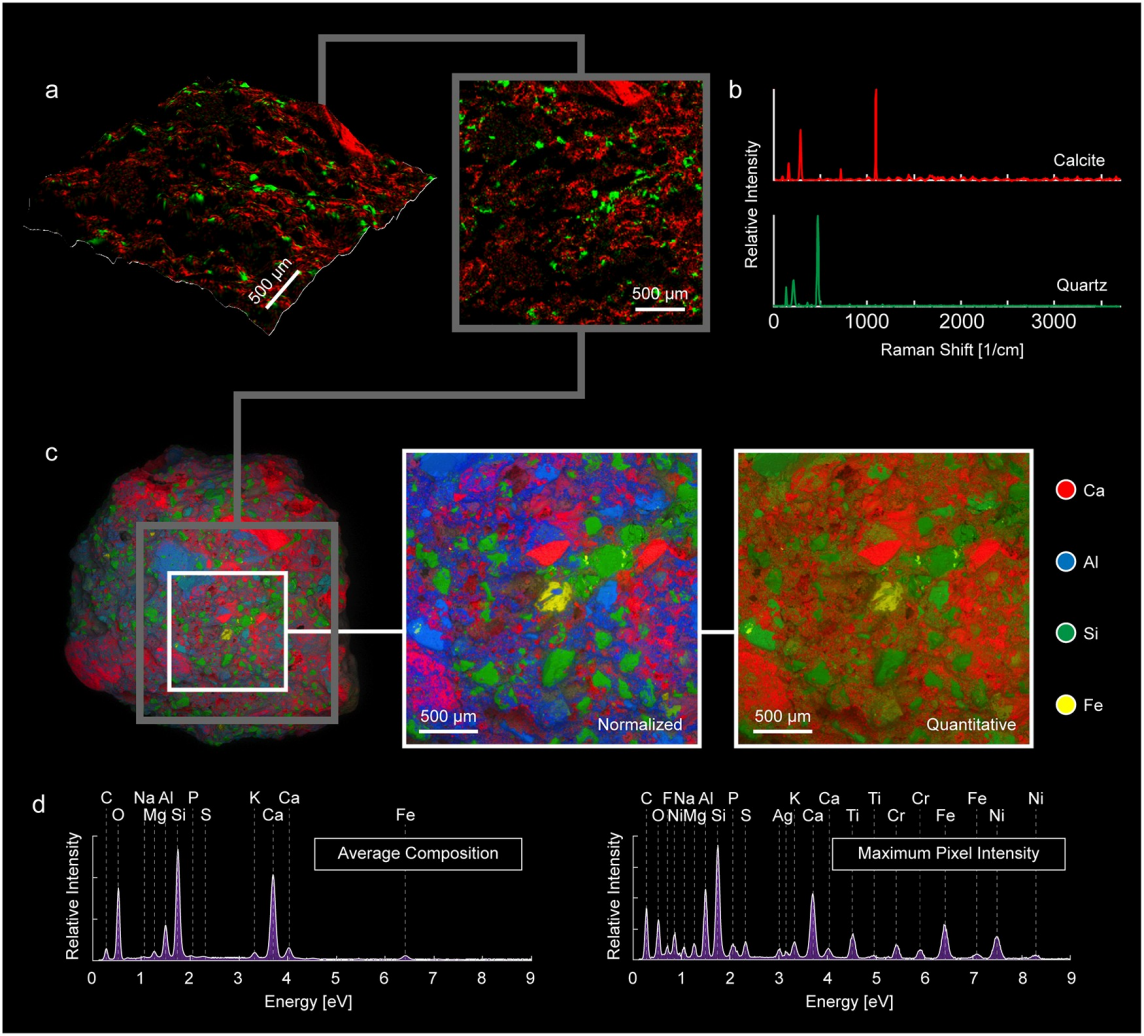
Correlative EDS and Raman characterization of the fractured surface of a sample of ancient Roman concrete. (a) 3D and plan views of Raman phase map, (b) constituent Raman spectra (color-coded corresponding to the different displayed map phases). (c) EDS element map of the polished concrete sample, with normalized and quantitative element maps of region within white square. (d) EDS spectra for the entire element map indicating the total average composition of the sample (left) and the combined maximum pixel intensities of the detected elements (right).

EDS and Raman spectroscopy were also used to generate phase maps of the fracture surface of an ancient Roman concrete sample from the same region of the perimeter wall. Optically, there is a much lower proportion of aggregate visible on the fracture surface of the concrete than in the cross section. Therefore, data collected on the fracture surface is expected to be more representative of the cementitious binding phase, whereas data collected for the polished thin section would provide more information about the average composition, including the aggregate composition, of the concrete. Fig 5a and 5b show the Raman phase map of the fracture surface and the corresponding Raman spectra of the phases detected using the same color scheme: quartz in green and calcite in red. Although quartz aggregate and calcite are still observed, a much larger region of the measured area yields Raman spectra with high background fluorescence, supporting the assumption that the fracture surface contains a greater proportion of the cementitious binding phase.

Fig 5c shows both the quantitative and normalized EDS element maps obtained for the fracture surface. The corresponding total counts for the X-rays emitted at different energies and the maximum number of counts detected anywhere on the measured area, indicated by the white box on the sample in Fig 5c, are shown in the EDS spectra in Fig 5d. The Raman phase map (Fig 5a, right) of the region indicated by the grey box in Fig 5c, was created from the specific color-coded Raman spectra of the detected phases extracted from the total dataset using NMF in Fig 5b, which were then superimposed onto the surface topography data collected using *TrueSurface* profilometry, and shown in Fig 5a, left.

**Fig 6:**
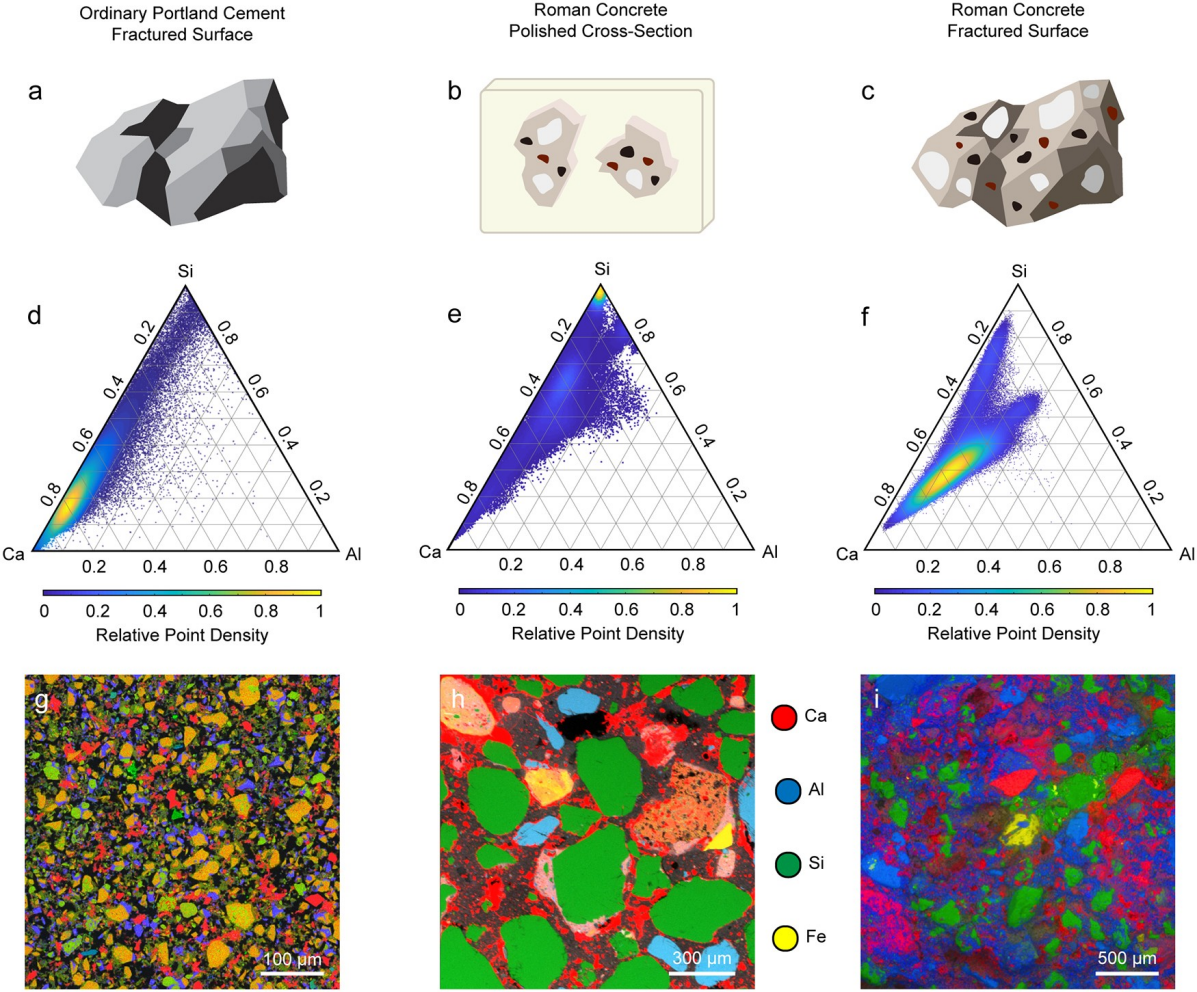
Ternary density plots and EDS element maps of ordinary Portland cement and ancient Roman concrete. Schematics of (a) the fractured surface of an ordinary Portland cement sample, (b) the polished cross-section of ancient Roman concrete that has been embedded in resin and (c) the fractured surface of an ancient Roman concrete sample. Below the sample illustrations (upper row) are the ternary density plots (middle row) showing ratios of Ca to Si to Al at each pixel in the different EDS maps (bottom row). For each of the ternary density plots, the purple regions are most sparsely populated, and the yellow regions are most densely populated.

EDS element maps were collected on the fractured surface of ordinary Portland cement and ancient Roman concrete and on the polished cross-section of resin-embedded ancient Roman concrete. Schematics of these samples and the corresponding normalized element maps are shown in the top and bottom rows on Fig 6, respectively. From each EDS dataset, a set of 16-bit greyscale quantitative images with pixel brightness values representing the atomic percentage of calcium, silicon and aluminum present at each pixel were generated. These quantitative images were used to plot the ratios of those elements relative to each other (i.e. Ca + Si + Al = 1) for all pixels in that sample’s dataset on ternary density plots. In each ternary density plot (Fig 6d, 6e and 6f), the ratios of calcium to silicon to aluminum at each pixel in the measured area is represented by a single point on the ternary diagram. To show the distribution of points on the ternary diagram more clearly, regions of the ternary diagram in which points are very densely distributed are shown in yellow, while sparse regions are shown in purple, as indicated by the color bars below the plots.

In ternary frequency diagrams, which have been used by other authors in the field of cement characterization to represent EDS data, the axes are divided into n bins, resulting in a 2D ternary histogram containing n^2^ bins. The ternary coordinates are then sorted into bins, and the number of coordinates in each bin is shown using an intuitive color map, which allows for the rapid identification of the major phases present.

**Fig 7:**
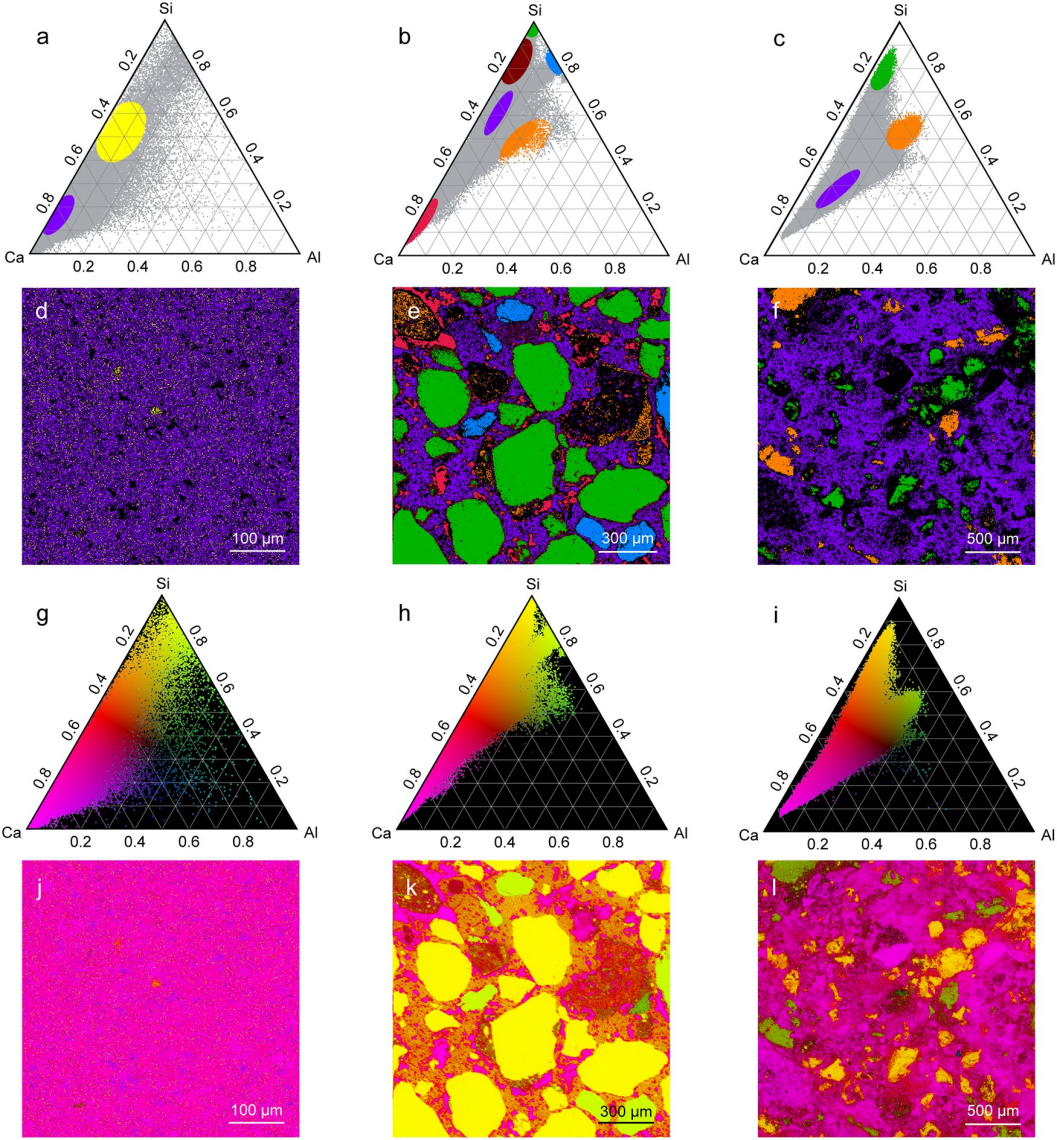
OPC and ancient Roman concrete phase identification and mapping. The leftmost column is the fractured surface of OPC, the middle column is the polished cross-section of ancient Roman concrete, and the rightmost column is the fractured surface of ancient Roman concrete. (a)-(c) All ternary coordinates are shown in grey, and points within particular ellipses are colored and their corresponding regions are shown in (d)-(f). (g)-(i) A color gradient is used to color each ternary coordinate based on its position in the ternary diagram, and its corresponding pixel is colored the same color in (j)-(l).

Fig 7 shows the implementation of two visualization tools that were developed for use in conjunction with the ternary density plots shown in Fig 6. In Fig 7a, 7b and 7c, sub-regions of the ternary density plots (denoted by ellipses) were selected which corresponded to specific elemental ratios of interest, and the sample regions corresponding with the ternary coordinates in those ellipses were colored with the same colors in Fig 7d, 7e and 7f. Fig 7g, 7h and 7i show another method of distinguishing different regions of the ternary diagram. In this technique, a color was chosen for each of the three vertices of the ternary diagram, and each ternary coordinate was colored using a mixture of the three colors corresponding to its location on the ternary diagram. The corresponding pixel from the EDS map was then colored using this same color space in order to simultaneously visualize all of the different phases present in the entire mapped sample (Fig 7j, 7k and 7l).

The ternary density plot for OPC (Fig 6d) shows most ternary coordinates lying close to the Ca/Si axis, with a very densely populated cluster close to the Ca vertex. This suggests a very large proportion of C-S-H and low-alumina C-A-S-H, which is expected for OPC [39]. The ternary density plot for the fracture surface of ancient Roman concrete (Fig 6f), however, shows coordinates clustered away from the Ca-Si axis. The large dense cluster corresponds with the region occupied by higher-alumina C-A-S-H, suggesting a much greater presence of this binding phase in the ancient Roman concrete sample than in modern cement. The “arm” of the ternary coordinates closer to the top of the ternary diagram corresponds with quartz that is partially covered with binder, which shifts its coordinates away from the Si vertex. This is corroborated by comparing the phase map in Fig 7f with the corresponding Raman phase map showing quartz distribution in Fig 5a. The arm of Fig 6f that extend towards the center of the ternary diagram corresponds to the region in which natural pozzolans would be found [40]; natural pozzolans in the form of volcanic ash was a common additive to ancient Roman concrete mixtures [5].

The ternary density plot for the polished cross-section of ancient Roman concrete provides more insight into the aggregates used in the production of this Roman concrete. The cluster at the very top of the ternary diagram close to the Si vertex corresponds to quartz aggregate. The region in the red ellipse in Fig 7b, which corresponds to the region in which C-S-H would be found, is found in the binder of the ancient Roman concrete cross-section, but it is absent from the fracture surface of ancient Roman concrete. Since a material would fracture along its weakest component, these results suggest that the higher-alumina C-A-S-H found on the fracture surface is weaker than the lower-alumina C-A-S-H and C-S-H that is more prevalent in the binding phase observed in the polished cross-section. Other aggregate found in the ancient Roman concrete that contains more aluminum is colored blue and shown in Fig 7b and 7e. The majority of the binder appears to be some form of C-A-S-H, but the binder that is located near the perimeter of the quartz aggregate is richer in silicon (colored burgundy in Fig 7b and 7e). Expectedly, natural pozzolans are also found in the polished cross-section of the ancient Roman concrete sample.

## Discussion and future work

Using Roman concrete as a model research system, we have presented a set of complementary high-resolution correlative tools for the characterization of cementitious materials. Primary phases in these samples were detected using large-area CRM phase mapping. For fractured samples with intrinsically irregular surface topographies, profilometry measurements were performed to generate a 3D surface map, which was then used in conjunction with the Raman microscope’s motorized piezo stage to maintain the constant intersection of the sample surface with the focal plane. This approach enabled the detection of a Raman signal over the entire area of interest, while maintaining confocality (Figs 2 and 5).

While Raman spectroscopy is extremely useful in identifying phases that consist of Raman active chemical bonds, this technique loses its utility when there are complex phases in materials that have no distinct stretching or bending modes that give a clear Raman shift. EDS, in contrast, is able to map the elements present on a sample’s surface, and while it cannot identify specific phases, it is still a very effective approach for obtaining compositional information for sample regions that cannot be successfully identified using Raman spectroscopy. In addition, the use of multiple EDS detectors for signal acquisition provides a relatively uniform coverage of samples with complex surface topographies, while largely minimizing shadowing artifacts that commonly occur when single detector systems are employed.

For the characterization of cementitious materials, identification of the mechanically weak interfaces, which can lead to catastrophic structural failure, is critical, and current efforts are underway to apply the techniques outlined here to investigate the composition of these components observed on the fracture surfaces and between the aggregate in the cross section, for both ancient roman concrete and contemporary OPC.

Although the focus of this work was on the characterization of ancient Roman concrete, the techniques and tools presented in this study could be applied to the characterization of a wide range complex, heterogeneous materials with irregular surfaces. In this work, EDS data was correlated with Raman phase maps, but this correlation could be extended to any two (or more) datasets for which an image may be generated. The large amount data that can be quickly obtained using EDS and the associated data processing tools described herein could be used for the statistical characterization of essentially any heterogeneous material of interest, and the advanced large-area spectroscopic phase mapping techniques presented here show great potential in using advanced computational analysis in the study of macroscopic structures.

## Materials and methods

Ancient Roman concrete samples were collected from various walls from the Privernum archaeological site with permission and supervision from the director of the Sistema Museale Priverno, Margherita Cancellieri, and the regional official of the Ministero per I Beni Culturali e Ambientali, Nicoletta Cassieri. Work at the archaeological area did not involve endangered or protected species, and no permits were required for the described study. All samples discussed in this work are stored in the Laboratory for Multiscale Characterization and Materials Design at the Massachusetts Institute of Technology.

Samples in their original state were imaged using energy-dispersive X-ray spectroscopy, backscattered electron microscopy, *TrueSurface* profilometry, and confocal Raman microscopy. These imaging techniques were also performed on polished resin-embedded thin sections, which were prepared as follows. The concrete samples were first embedded in a cold-cure epoxy and trimmed to size with a slow speed water-cooled diamond saw. The resulting samples were then progressively polished with diamond grinding disks from 60 grit to 800 grit, followed by final polishing using diamond suspensions down to 0.25 μm.

Multi-detector EDS was performed using a Tescan Vega GMU scanning electron microscope equipped with two Bruker XFlash 5030 X-ray detectors. Mapping data were acquired at a 15mm analytical working distance at an accelerating voltage of 20keV. Mapping times varied as a function of map pixel density as well as the specific elemental abundances in the phases being analyzed, but for all of the Ca to Si to Al phase diagrams generated in this work, acquisition times were always determined such that they resulted in a minimum of 5000 counts per pixel. The acquired spectra from each pixel were then used to calculate elemental ratios, which were then exported as single element quant maps and used for generation of the ternary diagrams.

For the Raman phase mapping of topographically complex fractured surfaces, surface profilometry measurements were conducted using WITec’s *TrueSurface* microscopy module, which maps three-dimensional surfaces using chromatic aberration techniques. A beam of white light is directed at the surface of the sample; since the components of this beam of white light have different focal distances, the elevation of a location on the sample may be deduced when the CCD camera detects a particular color. After measuring the topography, large-area CRM mapping was then executed and the resulting phase map collected.

Raman phase mapping was conducted using a confocal Raman microscope system (alpha300R, WITec, Ulm, Germany). The system consisted of a frequency-doubled Nd:YAG 532 nm laser, which was used along with a multi-axis piezo scanner (P-527, Physik Instrumente, Karlsruhe, Germany) and a motorized large-area stage for sample positioning. As the sample was scanned, the system used the topographic information collected using *TrueSurface* profilometry to maintain confocality during large area mapping. Raman spectra were acquired using a thermoelectrically cooled CCD detector (DU401A-BV, Andor, UK) placed behind the spectrometer (UHTS 300, WITec, Ulm, Germany) using a grating of 600 g mm^-1^.

Raman phase mapping was carried out at one-third maximum intensity to avoid damage to the sample. For each point on the sample at which a Raman spectrum was recorded, an integration time of 300 ms was used, and at each pixel, three spectra were recorded, and the average was taken. For each sample, an area of 8000 μm by 8000 μm was measured at a resolution of 200 pixels by 200 pixels. WITec Control FOUR (version 4.1, Witec) was used for data acquisition, and WITec Project FOUR (version 4.1, Witec) was used for cosmic ray removal, smoothing and background subtraction, as well as for conducting NMF to determine the spectra of individual phases contained within the total set of spectra collected during large area scans.

## Abbreviations

ASR: Alkali silica reaction
BS-SEM: Backscattered scanning electron microscopy
C-A-S-H: Calcium alumina silicate hydrate
CH: Portlandite
C-N-A-S-H: Calcium sodium alumina silicate hydrate
CRM: Confocal Raman microscopy
C-S-H: Calcium silicate hydrate
EDS: Energy-dispersive spectroscopy
NMF: Non-negative matrix factorization
OPC: Ordinary Portland cement
SEM: Scanning electron microscopy
